# Development of self-healing model of rock fracture for PFC simulation

**DOI:** 10.1128/spectrum.03809-25

**Published:** 2026-07-22

**Authors:** Yingying Hu, Jifan Liu, Dehu Wang, Weitao Liu, Shuai Zhang

**Affiliations:** 1 Shandong Key Laboratory of Eco-Environmental Science for the Yellow River Delta, Shandong University of Aeronautics, Bin zhou, China; 2 College of Energy and Mining Engineering, Shandong University of Science and Technology, Qingdao, China; Nanjing Agricultural University, Nanjing, China

**Keywords:** rock fracture bioremediation, microbial biomineralization, MICP-coupled numerical simulation, urea hydrolysis, biogeotechnology, *Bacillus pasteurii*

## Abstract

Rock mass fractures often lead to structural instability, posing significant safety risks in underground engineering such as mines and tunnels, including water seepage, air leakage, and damage to concrete structures. This study aims to enhance grouting-based repair techniques by investigating the mechanisms of microbially induced calcium carbonate precipitation (MICP) and simulating fracture filling processes. Laboratory experiments were conducted to identify optimal culture conditions for maximizing calcium carbonate production. Additionally, Particle Flow Code (PFC) 5.0 was employed to simulate the filling of fractures with microbial calcium carbonate. The results indicate that urea, urease-producing bacteria, and ammonium ions exhibit continuous concentration distributions within fractures, regulated by chemical reaction kinetics. *Bacillus pasteurii* demonstrated the highest calcium carbonate yield under conditions of pH 9, urea concentration of 1–1.5 mol/L, and calcium ion concentration of 1 mol/L. Microbial calcium carbonate filling significantly improved the load-bearing capacity of fractured rock masses. However, the simulation has certain limitations, including the inability to model dynamic changes in reaction rates and product distribution, and the assumption of maximum calcium carbonate filling for analysis. Despite these constraints, the integrated experimental and numerical approach reliably captures the fracture-healing behavior driven by MICP. These findings provide critical theoretical support for optimizing microbial culture conditions, regulating reactant concentrations, and controlling pH to improve the efficiency of fractured rock repair and ensure the long-term safety of underground engineering structures.

**IMPORTANCE:**

Cracks in rocks and concrete structures pose significant threats to the safety and longevity of underground engineering projects, such as mines and tunnels, leading to water leakage and instability. Traditional repair methods are often costly, disruptive, and environmentally taxing. This study harnesses the power of bacteria to create a self-healing material, effectively using microbes to produce a natural bio-cement that seals these dangerous fractures. By identifying the optimal conditions for the bacteria to work and developing a computer model to predict the healing process, our research provides a ground-breaking, eco-friendly strategy for autonomous infrastructure repair. This bio-based approach not only enhances engineering safety but also paves the way for sustainable construction practices, turning a biological process into a powerful engineering solution.

## INTRODUCTION

Microbially induced calcium carbonate precipitation (MICP) has emerged as a promising biogeotechnical approach with diverse applications, including mine ventilation sealing, self-healing of concrete cracks, and soft ground stabilization (1–3). Conventional techniques for addressing these issues typically involve mechanical processing or the use of synthetic materials, which are often costly, energy-intensive, and environmentally invasive.

When urea and calcium ions are introduced into the culture environment of urease-active bacteria such as *Bacillus pasteurii* or *Bacillus sphaericus*, these microorganisms can rapidly induce calcium carbonate precipitation. This property holds significant potential for sealing fractures in rock masses and controlling seepage in the context of microbial seepage prevention (4). Previous studies have explored various factors influencing MICP efficiency. For instance, Kim et al. (1) investigated the effects of temperature, activation time, and pH on urease activity in the context of MICP. Increasing bacterial concentration and metabolic activity enhances calcium carbonate formation, which, in turn, improves the internal friction angle and cohesion of the treated material, thereby reducing fracture permeability and restoring mechanical strength (1–3). After 5 days, 50 μm rhombic calcite crystals were detected on the crack surface of the lime specimen, and the overall weight of the test specimen increased by 0.3–1.1 g (4). Compared with an untreated lime sample, the water permeability of the test group sample decreased significantly (4, 5). Previous studies demonstrated the use of microbial calcium carbonate as a mineral plugging agent in oilfield sandstone, achieving a notable reduction in permeability (4–7). Subsequent studies have developed diffusion models for microbial grouting in fractured media, incorporating parameters such as bacterial concentration, calcium ion concentration, and urea concentration to assess permeability changes in rock masses (4, 8, 9). These models also establish a directly proportional relationship between fracture width and mineral deposition. Previous studies have consistently demonstrated the advantages of microbial grouting, including low viscosity and low-pressure injection (10), improved hydraulic properties of rock joints (11), and significant reductions in permeability coefficients of rock fractures in submerged environments (12).

In the grouting process, there are many influencing factors, including pH, temperature, nutrient concentration, calcium ion type, urea concentration, bacterial liquid concentration, etc. The suitable pH range for urease activity is 7–10; values outside this range inhibit the enzyme (13–18). Similarly, high Ca²^+^ concentrations adversely affect urea hydrolysis, while low concentrations reduce calcium carbonate precipitation (19). The team at Tsinghua University studied the anti-liquefaction ability of sand foundations reinforced by microbial grouting. Through shaking table model tests, it is explained that the seismic performance and anti-liquefaction performance obtained by microbial grouting technology are better than the corresponding performance after grouting with traditional technology (20).

Despite these advances, most studies have focused on applications such as self-healing concrete, wastewater treatment, and sand stabilization, with limited attention given to the use of MICP for sealing rock fractures (21, 22). Moreover, the distribution of grouting materials within fractures is affected by fracture width, surface roughness, permeability, and tortuosity (23), which impose additional constraints on microbial viability and the uniformity of calcium carbonate precipitation (24). The underlying mechanisms of microbial grouting in fractured rock remain underexplored. To date, no previous study has employed Particle Flow Code (PFC) simulation to model the dynamic process of MICP-based fracture repair, including the effects of calcium carbonate filling on mechanical behavior. Furthermore, the integration of systematically optimized culture conditions with discrete element modeling represents a novel approach to quantify the relationship between bacterial growth parameters and macroscopic load-bearing capacity. In this paper, based on the microbial slurry and fractured rock mass characteristics, using a simulation experiment, we build a reasonable model by adjusting various factors to compare the continuous distribution of urea, urease-producing bacteria, and ammonium ions in the fractures. Furthermore, we study the relationship between the precipitation rate of calcium carbonate crystals and the characteristics of rock fractures in order to better observe the dynamic process and grouting effect of microbial grouting.

## MATERIALS AND METHODS

**Fig 1:**
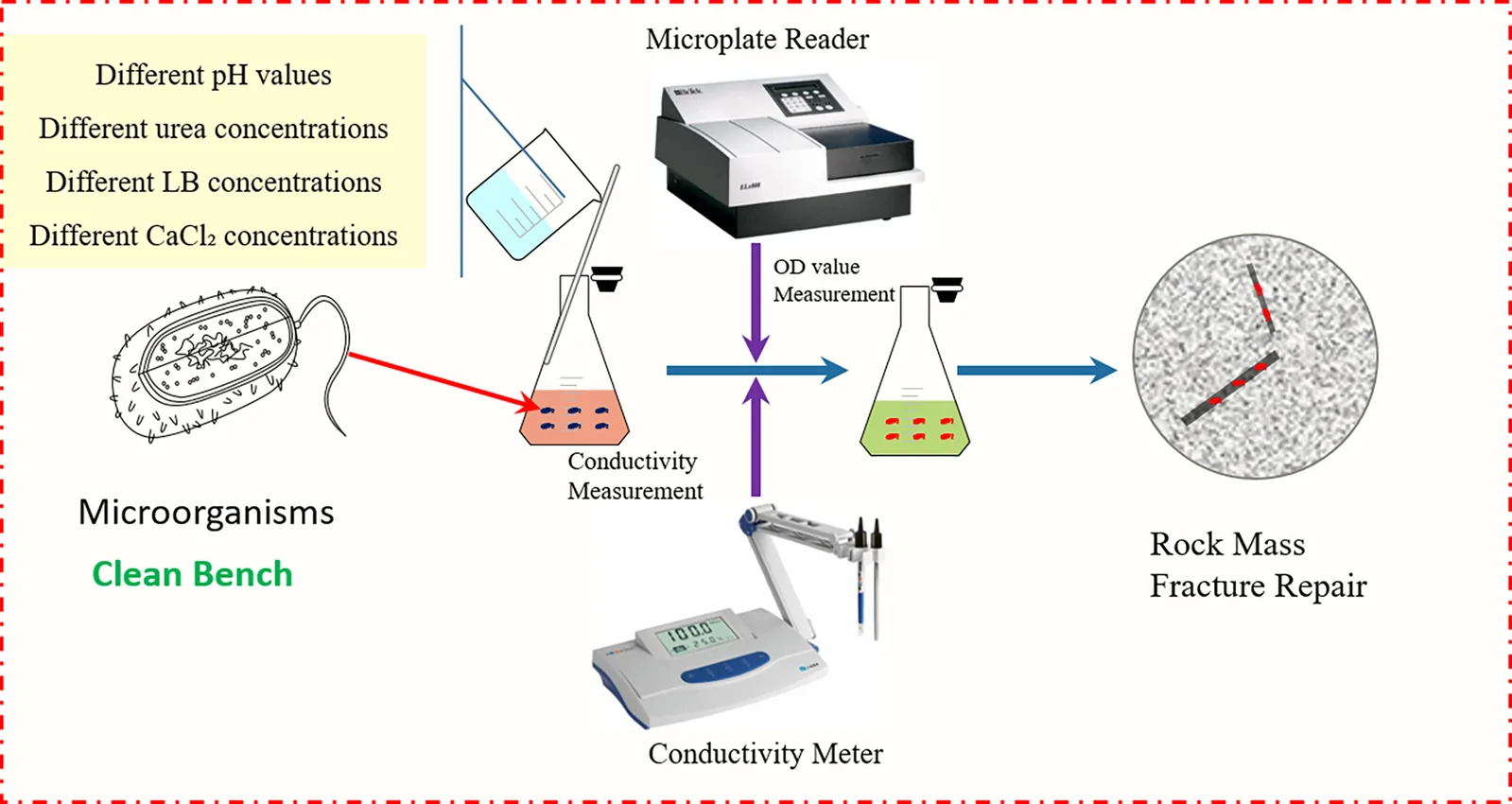
Schematic diagram of the overall research approach, illustrating the integration of microbial experiments and PFC numerical simulation.

**Fig 2:**
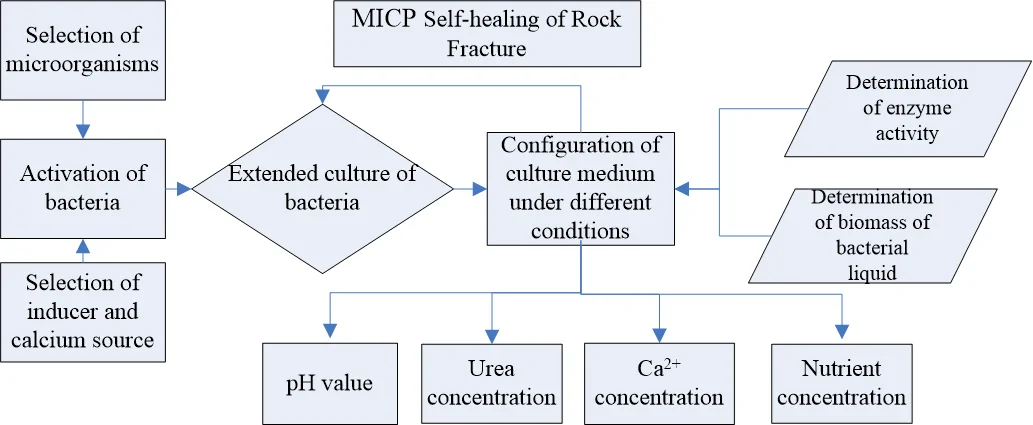
Experimental flowchart detailing the steps of bacterial culture, medium configuration, and calcium carbonate precipitation induction.

The crystal form and quality of calcium carbonate induced by *Bacillus pasteurii* and *Bacillus sphaericus* are mainly controlled by various factors in the microbial experiment, such as the microorganism type, calcium source, pH value of nutrient solution, and urea concentration. All procedures were performed under aseptic conditions. The experimental design is illustrated in Fig. 1 and 2. The sequential steps were (i) activation of freeze-dried bacteria in LB broth; (ii) expansion culture (28°C, 200 rpm, 24 h); (iii) preparation of media with varying pH (7–12), urea (0–1.5 mol/L), Ca²^+^ (0–1.0 mol/L), and LB (5–30 g/L) following a one-variable-at-a-time approach; (iv) inoculation (1%, vol/vol) and incubation (24 h); (v) measurement of biomass (OD600) and urease activity (conductivity method); (vi) induction of calcium carbonate precipitation; (vii) PFC simulation. All experiments were performed in triplicate; data are presented as mean ± standard deviation (SD).

### Bacterial strains and culture media

#### Chemicals

The chemicals used in this experiment are listed in Table S1 available at https://doi.org/10.5281/zenodo.20840432. All reagents were of analytical grade unless otherwise specified. The main instruments used in this study are listed in Table S2 available at https://doi.org/10.5281/zenodo.20840432.

Grouted rock specimens were fabricated as cylindrical samples with a diameter of 50 mm and a height of 100 mm, and artificial fractures were pre-generated via the Brazilian splitting test method (a standard technique for inducing tensile fractures in rock mechanics). As presented in Table 1, the target rock type is sandstone—a typical sedimentary rock composed primarily of sand-sized grains (e.g., quartz, feldspar) cemented by materials such as clay minerals or carbonates. This rock type is widely distributed across diverse geological settings and engineering contexts, including building foundations, tunnel surrounding rocks, and slope rock masses (25).

**TABLE 1:** Characteristics of grouting samples

Rock type	Rock density (g/cm³）	Porosity	Deformation modulus (10⁴ MPa）	Compressive strength (MPa）	Tensile strength (MPa）	Cohesion C	Friction angle
Sandstone	2.2–2.71	1.6–28	6.7–17.2	20–100	4–25	8–40	35°−50°

Notably, the fracture characteristics of sandstone are strongly dependent on its porosity: sandstones with high porosity exhibit abundant primary pores, which act as preferential pathways for fracture initiation and propagation, resulting in fractures typically characterized by a reticulated or dispersed pattern. In contrast, low-porosity sandstones tend to develop more concentrated fractures, which predominantly propagate along weak grain-cementation interfaces or mineral boundaries.

Mechanical properties further govern fracture behavior: sandstones with low compressive strength undergo rapid fracture propagation and coalescence under axial compression, while those with low tensile strength are susceptible to tensile fracture extension when exposed to tensile stresses (e.g., groundwater seepage pressure, blasting-induced disturbance). Additionally, sandstones with low cohesion (C) and small internal friction angles exhibit poor anti-sliding capacity along fracture surfaces. In engineering practice, the presence of bedding-parallel fractures or weak interlayers in such sandstones significantly increases the risk of landslides and collapses (26–28).

### Selection of inducer and calcium source

Because of calcium carbonate deposition induced by microorganisms, urea can be hydrolysed in solution by urease as the inducer of the microbial growth environment. Due to the hydrolysis of urea, the pH value of the reaction solution increases continuously. Then, as a result of a series of biochemical reactions, a large number of carbonate ions are produced, which are important for the formation of calcium carbonate precipitates. Anhydrous calcium chloride was selected as the calcium source due to its high solubility in water (approximately 74.5 g per 100 g water at 20°C), which facilitates the preparation of high-concentration calcium solutions required for efficient carbonate precipitation. The reaction mechanism of MICP is as follows (29):


$$Ca^{2+}+Cell\to Cell-Ca^{2+}$$

$$CO(NH_{2}{)}_{2}+H_{2}O\to NH_{3}+CO_{2}$$

$$NH_{3}+H_{2}O\to NH_{4}^{+}+OH^{-}$$

$$CO_{2}+OH^{-}\to HCO_{3}^{-}$$

$$Cl^{-}+HCO_{3}^{-}+NH_{3}↔NH_{4}Cl+CO_{3}^{2-}$$

$$Cell-Ca^{2+}+CO_{3}^{2-}\to Cell-CaCO_{3}$$

According to the above reactions, increasing calcium ion and urea concentrations can theoretically enhance the MICP reaction rate and calcium carbonate crystallization. However, previous studies have shown that both urea and Ca²^+^ concentrations exhibit optimal ranges (typically 0.5–1.0 mol/L for urea and 0.5–1.0 mol/L for Ca²^+^), beyond which urease activity is inhibited (19).

### Activation and expansion culture

Bacterial activation and expansion were carried out under aseptic conditions.

#### Activation

Freeze-dried *Bacillus pasteurii* (Guangdong Microbial Culture Collection Center) was rehydrated in 5 mL of sterile LB broth (10 g/L tryptone, 5 g/L yeast extract, 10 g/L NaCl, pH 7.0) and incubated at 28°C ± 0.5°C with orbital shaking at 200 rpm for 24 h.

#### Expansion culture

**Fig 3:**
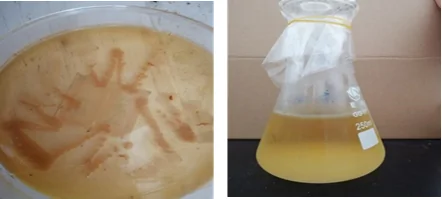
Activation and expansion culture of bacterial strains under sterile conditions

**Fig 4:**
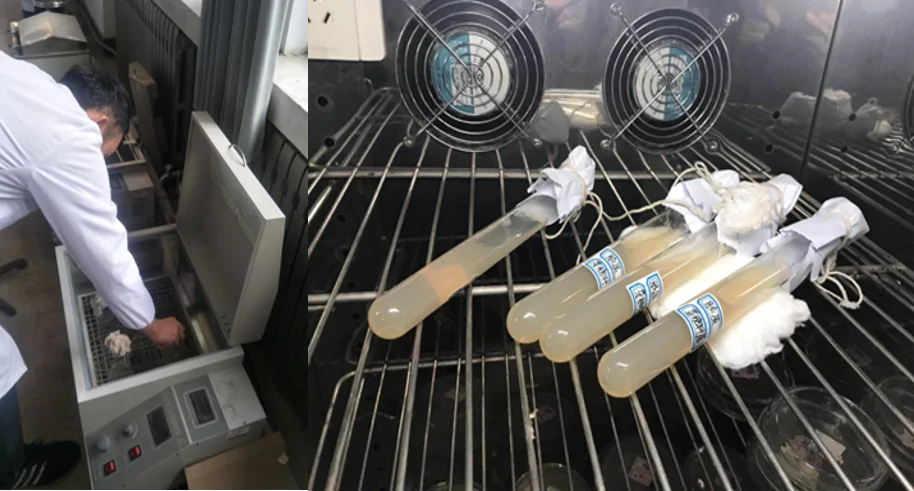
Bacterial cultivation in a constant temperature oscillation chamber at 200 rpm and 28°C.

For initial activation and expansion, LB broth was prepared at 25 g/L (5 g LB powder in 200 mL distilled water), following the manufacturer’s standard recipe. A 1 mL aliquot of the activated culture (OD600 = 0.8–1.0) was transferred to 200 mL of fresh LB broth (pH 8.0) and incubated under the same conditions for an additional 24 h. Bacterial growth was monitored by measuring OD600 every 2 h using a spectrophotometer (723PC, Chengdu Yike). To relate optical density to cell concentration, a calibration curve was established by serial dilution and plate counting on LB agar. Under our culture conditions (LB broth, pH 8.0, 28°C), an OD600 of 1.0 corresponded to approximately 1.2 × 10⁸ CFU/mL. This correlation was used to ensure consistent bacterial dosage across all experiments. The activated and expanded culture of the strain is shown in Fig. 3 and 4.

### Optimization of culture conditions (one-variable-at-a-time)

To systematically evaluate the effects of individual factors on bacterial growth and calcium carbonate precipitation, a one-variable-at-a-time approach was adopted. For each optimization experiment, all other cultural parameters were maintained at constant baseline levels, specifically: pH 8, urea concentration = 1.0 mol/L, Ca²^+^ concentration = 1.0 mol/L, and LB medium concentration = 20 g/L unless otherwise specified. These baseline conditions were established based on preliminary tests and literature values (13–18). Notably, the baseline LB concentration for optimization was set to 20 g/L (different from the 25 g/L used for initial activation), as preliminary experiments indicated that 20 g/L supported higher bacterial growth and calcium carbonate precipitation.

#### Optimization of pH value

A total of 1,300 mL of liquid medium was prepared, and 200 mL aliquots were aseptically dispensed into each of 6 conical flasks. All flasks contained the baseline concentrations of urea (1.0 mol/L), Ca²^+^ (1.0 mol/L), and LB medium (20 g/L). The pH of the medium in each flask was adjusted aseptically to target values (7, 8, 9, 10, 11, and 12) using sterile 1 M NaOH or HCl solutions, with the pH being monitored by a calibrated laboratory pH meter (Model: PHS-30). After sterilization, each flask was inoculated on an ultra-clean bench with *Bacillus pasteurii* at an inoculation amount of 1% (vol/vol) of the medium volume. After 24 h of incubation under standard conditions (28°C, 200 rpm), the OD600 values were measured using a visible light spectrophotometer (Model 723PC) and an M200 PRO microplate reader. For bacterial cultures with OD600 values between 0.2 and 0.8, the obtained value was multiplied by the corresponding dilution factor to yield the actual OD600 value. Growth curves were plotted with time as the abscissa and OD600 as the ordinate. All optimization experiments were performed in triplicate, and the reported OD600 values represent the mean of three independent cultures.

#### Optimization of urea concentration

A total of 1,500 mL of liquid medium was prepared, and 200 mL aliquots were aseptically dispensed into each of 7 conical flasks. The pH of all flasks was adjusted to 8, and the concentrations of Ca²^+^ (1.0 mol/L) and LB medium (20 g/L) were kept constant. After sterilization, 5 mL of filter-sterilized urea solution (prepared by passing through a 0.22 μm syringe filter) was added to each bottle to achieve final concentrations of 0, 0.25, 0.75, 1.0, 1.25, and 1.5 mol/L, respectively. A control group without urea (0 mol/L) was also included. Then, the cells were inoculated on an ultra-clean bench at an inoculation amount of 1% (vol/vol) of the medium volume. After 24 h of incubation, the OD600 values under different urea concentrations were measured. Curves were plotted with urea concentration as the abscissa and OD600 value as the ordinate. All optimization experiments were performed in triplicate, and the reported OD600 values represent the mean of three independent cultures.

#### Optimization of Ca²^+^ concentration

A total of 1,300 mL of liquid medium was prepared, and 200 mL aliquots were distributed into 6 conical flasks. The pH was adjusted to 8, and the urea concentration was fixed at 1.0 mol/L, while the LB medium concentration was maintained at 20 g/L. Anhydrous calcium chloride was added directly to each flask to achieve final concentrations of 0, 0.20, 0.40, 0.60, 0.80, and 1.00 mol/L, respectively. After sterilization, cultures were inoculated in a laminar flow hood at a rate of 1% (vol/vol) of the medium volume. After 24 h of incubation, the OD600 values under different Ca²^+^ concentrations were determined. Curves were plotted with Ca²^+^ concentration as the abscissa and OD600 value as the ordinate. All optimization experiments were performed in triplicate, and the reported OD600 values represent the mean of three independent cultures.

#### Optimization of nutrient concentration

LB (Lysogeny Broth) Broth Medium was prepared at concentrations of 5, 10, 15, 20, 25, and 30 g/L. For each concentration, 200 mL of medium was prepared, and the pH was adjusted to 8. The urea and Ca²^+^ concentrations were fixed at 1.0 mol/L each. After sterilization, each 200 mL LB medium was inoculated on an ultra-clean working table at an inoculation amount of 1% (vol/vol) of the medium volume. After 24 h of incubation, the OD600 values under different LB medium concentrations were determined. Curves were plotted with LB medium concentration as the abscissa and OD600 value as the ordinate. All optimization experiments were performed in triplicate, and the reported OD600 values represent the mean of three independent cultures.

### Biomass and urease activity measurements

#### Determination of biomass of bacterial liquid

Bacterial biomass was determined turbidimetrically by measuring optical density at 600 nm (OD600) using a visible spectrophotometer (Model 723PC). If the OD600 value of the bacterial liquid exceeds 0.8, it is necessary to dilute the bacterial liquid by a certain multiple so that the OD600 value of the diluted bacterial liquid is between 0.2 and 0.8, and then the OD600 value is measured. The obtained value multiplied by the corresponding dilution multiple is the actual OD600 value of the bacterial liquid. The prepared bacterial solutions under different conditions were placed in a constant temperature oscillation incubator for cultivation (30). The OD600 value was measured every 1 h for 24 h in each group to construct biomass growth curves (see Results section).

#### Determination of urease enzyme activity

The principle of measuring urease activity using conductivity is based on the hydrolysis of urea into ammonium and bicarbonate ions by urease, which increases the solution’s electrical conductivity. This change directly reflects enzyme activity. Baseline conductivity (*G*_0_) of the urea solution was measured. After adding the bacterial solution, conductivity changes were continuously monitored over time. The rate of urea consumption was then determined by converting the conductivity change using the established standard curve. Urease enzyme activity was calculated, where 1 U corresponds to the hydrolysis of 1 μmol of urea per minute (31).

After completion of bacterial culture, the enzyme activity of the bacterial liquid was measured using a conductivity meter (Model: DDS-11A). A 45 mL urea solution (1.1 mol/L) was prepared, and 5 mL of bacterial solution was added and mixed evenly. This 45 mL:5 mL ratio and 1.1 mol/L urea concentration were chosen to balance substrate availability, enzyme loading, and measurement precision, adhering to established principles in enzyme kinetics and conductivity-based assays (31). The electrode was immersed in the mixture, and after stabilization (fluctuation ≤0.1 μS/cm), the baseline conductivity (*G*_0_) was recorded. The change in conductivity of the solution was then measured over a 5-min period. A standard curve for urea consumption was prepared using known urease concentrations (0.1–10 U/mL). Each measurement was performed in triplicate. Prior to measurement, the electrode was rinsed with deionized water and the mixture was ensured to be at the experimental temperature (30°C ± 0.1°C).

#### Selection of microorganisms

Selecting and cultivating mineralized bacteria with strong adaptability and metabolism ability is the primary focus of MICP technology, and the crystal form, quality, and quantity of calcium carbonate crystals required are also closely related to this technology. In the microbial experiment, two kinds of bacteria, *Bacillus sphaericus* and *Bacillus pasteurii*, were selected in this paper because they have the following advantages:

Alkali resistance: In rock fractures, the external environment of mineralized bacteria is relatively harsh, its alkalinity is strong, and its pH value is approximately 11–13, which increases the alkali resistance of mineralized bacteria.

Environmental protection: Environmentally friendly environmental protection materials are important for microbial mineralization. The selected mineralized bacteria should cause as little pollution to the environment as possible and not pose a threat to human health.

**Fig 5:**
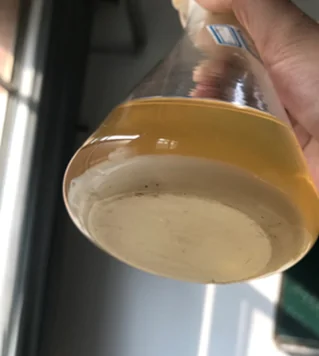
Microscopic image of calcium carbonate crystals formed by microbial mineralization.

Mineralization: Induction of microorganisms to produce calcium carbonate precipitation is used to prevent seepage in fractured rock masses. The most important product is calcium carbonate crystals, which have an ideal calcium carbonate crystal form (Fig. 5). Good quality and quantity of calcium carbonate are also important indicators to measure the quality and quality of the selected microorganisms.

### PFC numerical simulation setup

Numerical simulations were performed using PFC version 5.0. A two-dimensional discrete element model was constructed with dimensions of 100 mm × 100 mm, consisting of 15,060 particles with diameters ranging from 0.3 to 0.5 mm. The parallel bond model (PBM) was selected to simulate the cementation behavior between particles.

Model parameters were calibrated based on the mechanical properties of sandstone (25): deformation modulus = 5.0 × 10⁹ Pa, stiffness ratio (*k*_n_/*k*_s_) = 1.3, parallel bond modulus = 5.0 × 10⁹ Pa, tensile strength = 2.0 × 10⁶ Pa, cohesion = 5.0 × 10⁶ Pa, internal friction angle = 58°, and friction coefficient = 0.5. Each simulation was run with the same random seed to ensure reproducibility.

Pre-existing fractures were introduced into the model by systematically deleting selected parallel bonds along predefined planes or patterns, mimicking the typical tensile fracture patterns observed in the Brazilian splitting test of experimental sandstone specimens. The geometry of these fractures (e.g., single straight fracture, branched fracture) was designed to represent realistic rock discontinuities. For post-grouting simulations, calcium carbonate filling was modeled by introducing new particles with a deformation modulus of 1.0 × 10^9^ Pa (reflecting its lower stiffness compared to sandstone) into the fracture zones. These filling particles were either uniformly distributed or sparsely distributed within the fracture, depending on the simulation scenario (e.g., full filling vs partial filling). Uniaxial compression tests were simulated by applying a constant downward velocity of 0.01 m/s to the upper wall while the lower wall remained fixed and recording the axial force and displacement. A fixed timestep of 1.0 × 10⁻⁶ s was used to ensure numerical stability. Local non-viscous damping with a coefficient of 0.7 was applied. The simulation was terminated when the axial strain reached 5% or when the unbalanced force ratio fell below 1.0 × 10⁻⁵. The upper and lower walls moved at a constant velocity of 0.01 m/s under displacement control.

## RESULTS AND DISCUSSION

To explore the effect of sealing fractured rock masses in the subsequent grouting process and avoid the failure of preparatory work due to the lack of calcium carbonate crystals, appropriate bacterial strains were selected through a series of basic microbial experiments, where the culture conditions, including urea, CaCl₂, and nutrient concentrations, as well as pH, were systematically optimized. Through the introduction of PFC software, the interference factors that are not expected to appear in solving practical problems are minimized.

### Optimization of culture conditions

Table 2 shows that *Bacillus pasteurii* exhibits higher efficiency in catalyzing urea hydrolysis via urease, generating more NH₄^+^ and HCO₃⁻ ions in a shorter timeframe, thereby meeting the minimum requirement for enzyme activity in the experiment. The conductivity change of *Bacillus pasteurii* continues to increase with rising temperature, indicating that its urease activity enhances at higher temperatures, enabling adaptation to the thermal environment of high-temperature rock masses. The conductivity change of *Bacillus sphaericus* decreases at 40°C, indicating that its urease activity is inhibited at high temperatures and cannot function stably. Given that deep underground engineering (e.g., deep mines, geothermal reservoirs, and deep tunnels) experiences elevated rock temperatures due to geothermal gradients (typically 25–30°C/km), the thermal tolerance of *B. pasteurii* makes it a more suitable candidate for field-scale MICP applications in such environments. In contrast, *B. sphaericus* showed reduced activity at 40°C, indicating its unsuitability for deep-earth applications where temperatures may exceed this threshold. *Bacillus pasteurii* not only exhibits higher urease activity but also demonstrates enhanced activity and stability at elevated temperatures. It can adapt to high-temperature rock environments and sustain catalytic function, making it a more suitable experimental strain.

**TABLE 2:** Conductivity change (mS·cm⁻¹·min⁻¹) for urease activity measurement

Temperature/℃	20	30	40
*Bacillus pasteurii* conductivity change/mS· (cm·min)^−1^	0.19	0.24	0.27
*Bacillus sphaericus* conductivity change / mS· (cm·min)^−1^	0.11	0.16	0.12

The results of the microbial induction experiment laid a foundation for selecting suitable bacterial solutions in the following microbial grouting process.

**Fig 6:**
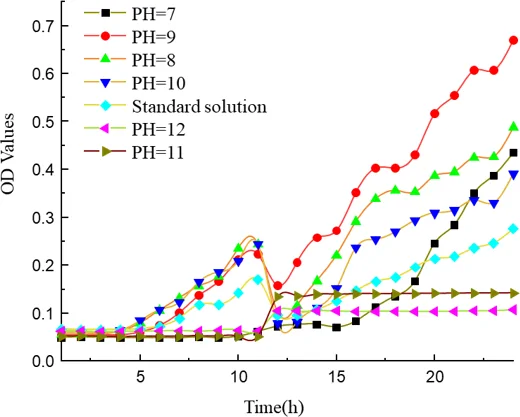
OD600 of *Bacillus pasteurii* at different pH values.

Figure 6 presents the growth curves of *Bacillus pasteurii* under different culture conditions. The growth patterns consistently exhibited four phases: lag phase 0–4 h, exponential phase 4–12 h, stationary phase 12–20 h, and decline phase after 20 h.

**Fig 7:**
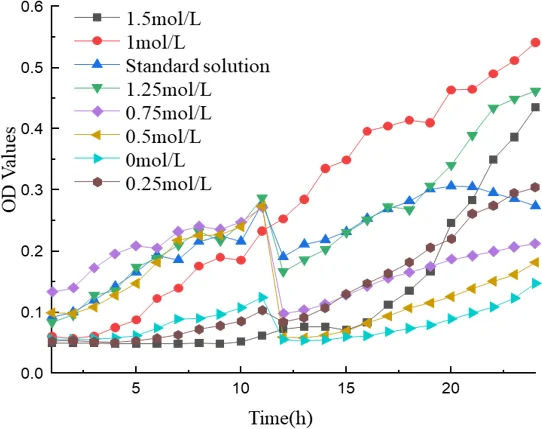
OD600 of *Bacillus pasteurii* at different urea concentrations.

**Fig 8:**
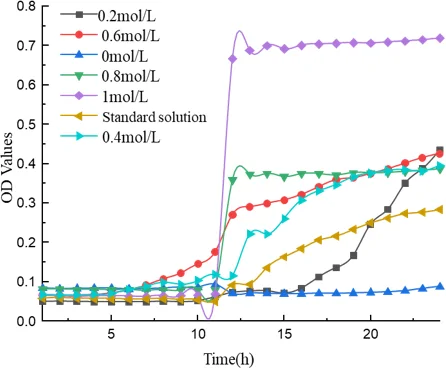
OD600 of *Bacillus pasteurii* at different Ca^2+^ concentrations.

**Fig 9:**
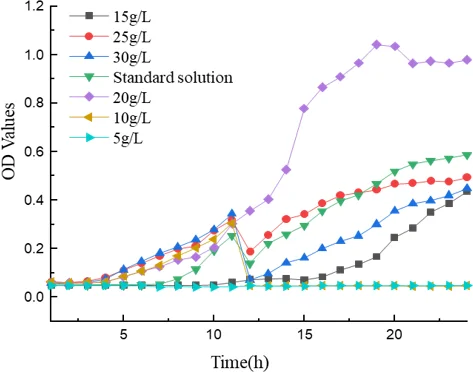
OD600 of *Bacillus pasteurii* for different nutrients.

The curve drawn from the test data in Fig. 6 shows that the biomass in the medium with pH values of 7, 8, 9, and 10 is higher than that of other cultures in the range of 5–10 h. After approximately 12 h, it continues to rise after a small decline. The final biomass of pH 9 in the four cultures is the largest. Therefore, the appropriate pH value of *Bacillus pasteurii* is 7–10. The pH is the best in the enclosure at 9. In Fig. 7, the biomass of the culture medium with urea concentrations of 1 mol/L, 1.25 mol/L, and 1.5 mol/L increased significantly after 10 h of culture time and was larger than that of other concentrations. Therefore, urea concentrations ranging from 1 to 1.5 mol/L have higher biological activity. In Fig. 8, there was no significant difference in the biological activity of the culture medium with different calcium concentrations before 10 h, but at 11 h, differentiation began. Except for the culture medium without calcium ions, the biomass of other culture media increased at a higher speed, and the biomass reached the maximum when the concentration of Ca^2+^ was 1 mol/L. According to the analysis of Fig. 9, when the LB concentration was adjusted to 20 g/L, the bacterial solution maintained the best biological activity. According to the analysis of the above charts, the microbial concentration of the expanded culture broth is the highest within 12–21 h, while the amount of calcium carbonate generated by other concentrations of culture medium is not ideal after a period of extended cultivation. Therefore, the bacterial solution can be selected for subsequent microbial grouting tests within the optimal concentration range of reactants obtained from the above analysis.

### PFC model calibration and validation

#### Methods and ideas

The model to be analyzed is constructed by PFC5.0. The ball contact model can select the parallel bonding model to load the specimen and carry out a uniaxial compression test. The mechanical properties of microbial grouting before and after filling with calcium carbonate are simulated, and the relationship between the strength change of the specimen before and after grouting is determined. This reflects the effect of microbial grouting on rock mass repair.

#### Parameter setting

First, the parameters of the model width in the coordinates are −0.05 m ~ 0.05 m and 10 cm × 10 cm. To ensure that the model is in line with the actual density of the concrete specimen, the particle diameter is set in the range of 0.0003 m ~ 0.0005 m, and the number of generated particles is 15,060 after running the command. In the contact model, the parallel bonding model is selected. This model has specific parameters and is a linear elastic bonding interface that can transfer force and torque, and the force chain distribution can also be displayed.

#### Basic introduction to the parallel bond model (PBM model)

The PBM simulates the cementation-like mechanical behavior (analogous to cement) at particle contact points. It assumes a finite-sized cementation zone between two contacting particles, where a set of elastic springs with constant normal stiffness (*k*ˉ*n*) and shear stiffness (*k*ˉ*s*) are uniformly distributed on the contact surface. These springs work synergistically with the linear contact springs of particles. The cementation zone can transmit normal force, shear force, and moment, which enriches the simulation of complex inter-particle mechanical responses. When simulating rock particle systems, the PBM effectively captures the cementation between mineral particles inside rocks, ensuring better consistency between simulation results and real-world behavior.

##### Mechanical mechanism of PBM model

Under external loads, relative displacements between particles induce forces and moments in the parallel bond zone. As the external load increases continuously, the parallel bond fractures when the tensile stress (*σ*ˉ) or shear stress (*τ*ˉ) in the bond zone exceeds the preset normal strength (*σ*ˉ*c*) or shear strength (*τ*ˉ*c*) of the bond, leading to microcrack initiation inside the material. The aggregation of numerous microcracks forms macro-cracks, eventually resulting in macroscopic failure of the material.

##### Applicability of PBM model

Simulation of brittle materials：The PBM exhibits significant advantages in simulating the mechanical behavior of brittle materials (e.g., rock and concrete). For brittle materials, inter-particle cementation strongly influences macroscopic properties. The PBM can capture the entire process of cementation from integrity to failure, thereby simulating the initiation, propagation, and final failure modes of cracks in brittle materials under loading. It provides reasonable simulation results for both the failure of intact rock under loading and the propagation of fractures in fractured rock under stress, supporting numerical analysis in rock engineering.

Simulation of complex stress states：The PBM effectively simulates material responses under complex stress states. Based on its mechanical mechanism, it accurately calculates the transmission and variation of inter-particle forces and moments, reflecting the deformation and failure characteristics of materials under different stress conditions.

##### Limitations of PBM model

Difficulty in parameter calibration：Accurate physical and mechanical parameters of cementation between mineral particles in rock cannot be directly measured through experiments. Consequently, the mesoscopic parameters of PFC models require repeated debugging and calibration.

Challenges in simulating complex geologic bodies：Natural complex geologic bodies exhibit high heterogeneity in internal structure and particle properties (spatial variations in mineral particle distribution and cementation degree in rock). Although the PBM can simulate heterogeneity to a certain extent, it remains challenging to accurately capture subtle spatial differences (32).

Inherent defect in strength simulation：The traditional PBM in PFC has an inherent defect: the simulated rock strength in tension-compression stress regions deviates from that of real brittle rocks (25, 26, 29, 31).

**Fig 10:**
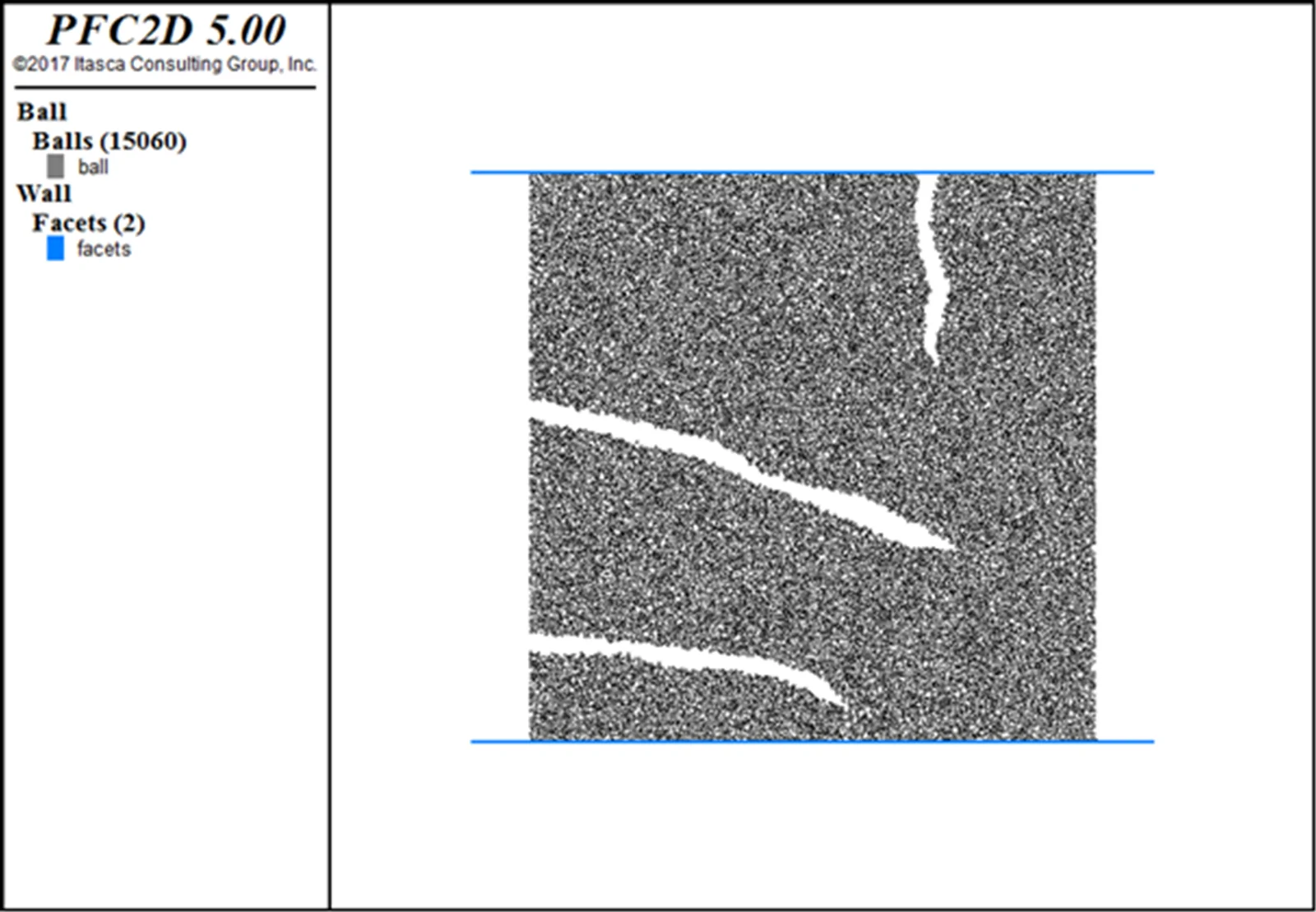
Discrete element model of the rock specimen with implemented parallel bonds, showing particle distribution and pre-existing fractures.

When the parallel bonding model is loaded with force, the resistance process is reflected as linear elasticity. When the force exceeds the strength limit that the model can bear, the parallel bonding model will be destroyed. When it is not bonded, it cannot transfer the force and moment. Second, the deformation modulus (deform emod) is set as 5.0e9 Pa, the stiffness ratio (the ratio of normal stiffness kn to tangential stiffness KS) is 1.3, and the parallel bond modulus (pb_deform emod) is set as 5.0e9 Pa. Tensile strength (pb_ten) is 2e6 Pa. The Cohesion (PB_ COH) was 5.0e6 Pa and internal friction angle (pb_fa) is 58°. The friction coefficient in the contact model is set as 0.5. The effect picture of the constructed model is shown in Fig. 10. In the process of filling cracks with calcium carbonate in the later stage, considering the different material properties of calcium carbonate and concrete, especially in strength, we can give calcium carbonate a lower stiffness value, which reflects the weak load capacity of calcium carbonate for concrete. Fig. 10 shows the discrete element model of the rock specimen with implemented parallel bonds. The model consisted of 15,060 particles with a porosity of approximately 16%, matching the typical porosity range of sandstone (1.6%–28%) (25). Pre-existing fractures were successfully introduced by deleting parallel bonds along predefined planes.

### Mechanical behavior before and after MICP treatment with numerical simulation

The test of the microbial grouting effect can be reflected by the mechanical properties of the specimen before and after repair. The uniaxial compression test is used to simulate the process, and the change law of the stress-strain curve and force displacement curve before and after repair is obtained. In the loading experiment, a 100 mm × 100 mm square model is used to simulate the loading process of PFC, and a two-dimensional model is used to simulate the whole process. Before filling, the loading surface of the specimen is continuously compressed.

**Fig 11:**
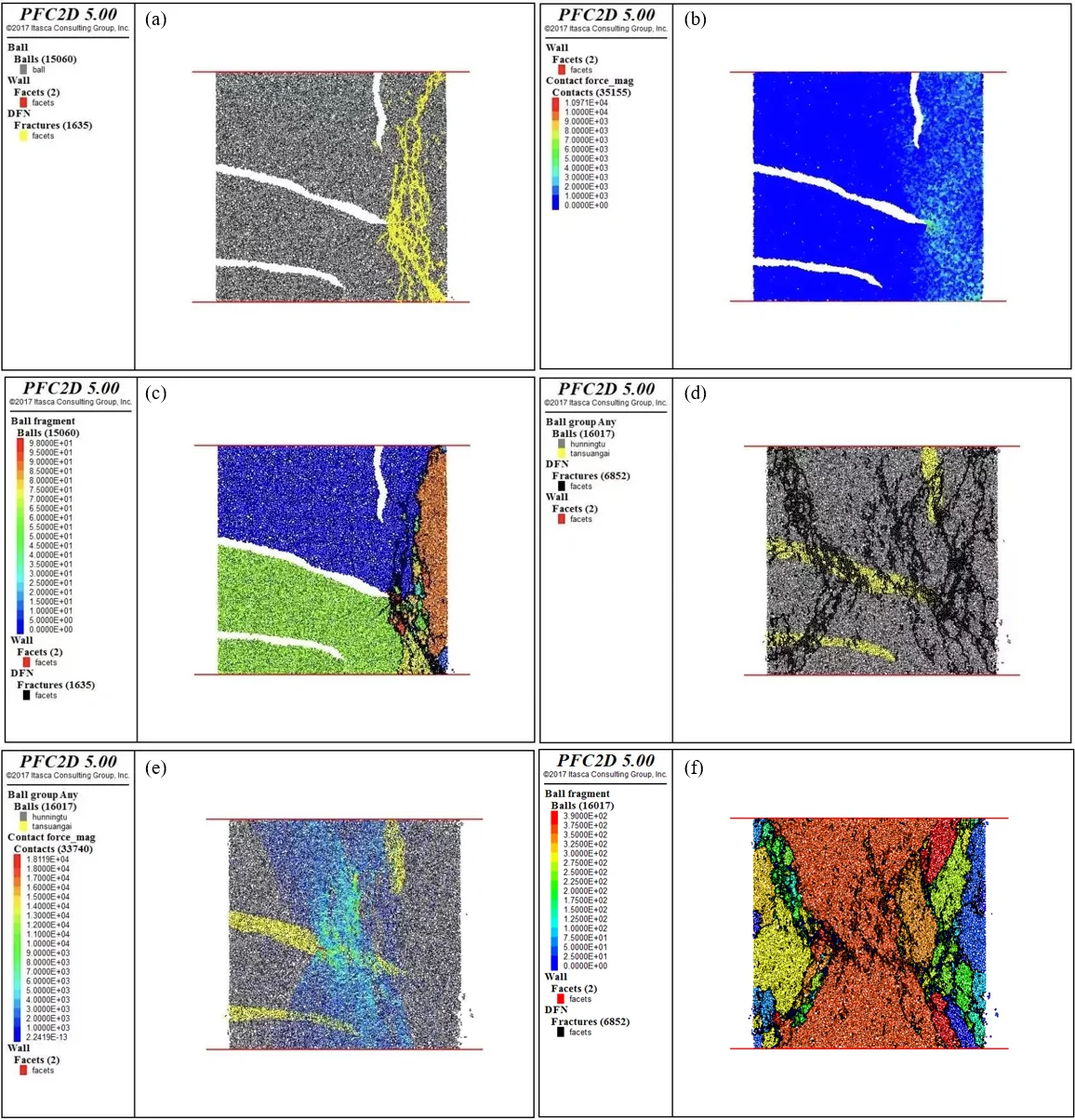
Numerical simulation results of uniaxial compression tests on fractured specimens before and after microbial grouting treatment: (**a**) crack distribution before filling; (**b**) force chain network before filling; (**c**) failure mode before filling; (**d**) crack distribution after filling; (**e**) force chain network after filling; (**f**) Final crack pattern after filling.

Before filling, the uniaxial compression test was simulated on the fractured specimen. As shown in Fig. 11a, the cracks generated during compression were mainly distributed in the regions with few pre-existing fractures. This occurs because areas with fewer cracks bear the primary load, making them more susceptible to new crack initiation under compression (25). After the compression test, the force chain distribution exhibited a pattern consistent with the original crack distribution: in regions with more fractures, the contact forces between particles were relatively low, whereas in intact areas, contact forces were higher, as illustrated in Fig. 11b. In addition, the specimen fractured into fragments of various sizes and shapes due to the applied load, as depicted in Fig. 11c.

After filling with calcium carbonate produced by microbial grouting, the uniaxial compression test was repeated. As shown in Fig. 11d, the crack distribution after compression became more uniform, indicating that the filling improved the stress distribution within the specimen. Furthermore, the force chain network (Fig. 11e) showed a significant change compared to that before filling: the region of high contact forces was concentrated in the middle part of the specimen, demonstrating a notable repair effect.

**Fig 12:**
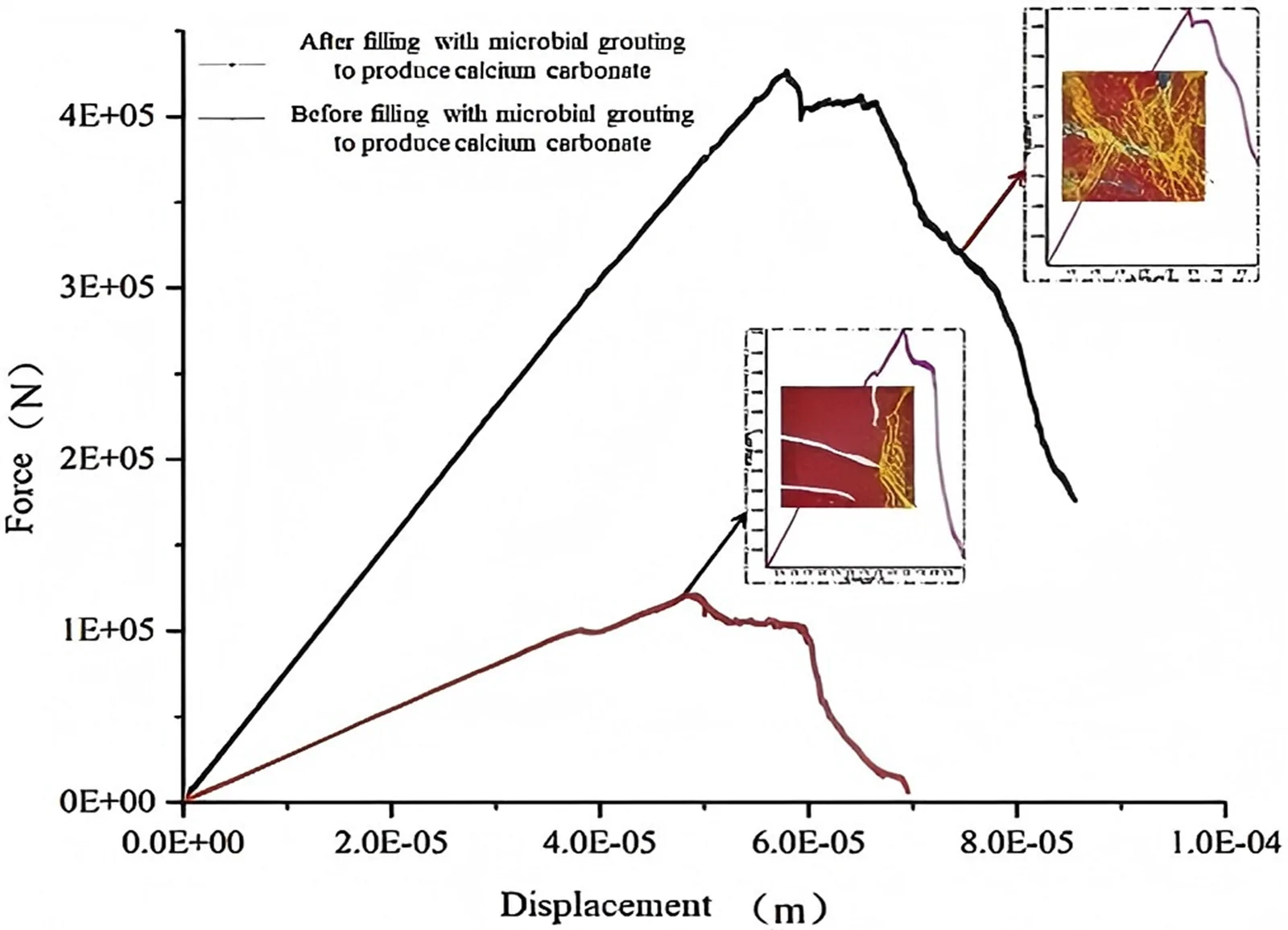
Force–displacement curves from uniaxial compression simulations before and after calcium carbonate filling, demonstrating a 3.28-fold improvement in load-bearing capacity.

Figure 12 presents the force–displacement curves obtained from the uniaxial compression simulations before and after calcium carbonate filling. The results show that the peak load increased from approximately 125 kN before filling to about 410 kN after filling, corresponding to a 3.28-fold improvement in load-bearing capacity. This indicates that microbial-induced calcium carbonate precipitation effectively enhances the mechanical properties of fractured rock masses. The stress-strain curve of the model can be easily obtained according to the force displacement curve because the model constructed in this chapter is a parallel bonding model, and the model size is 100 mm × 100 mm. Therefore, the value corresponding to the horizontal and vertical coordinates of the stress-strain curve only needs to be divided by 0.1 on the basis of the horizontal and vertical coordinates of the stress-strain curve. In addition, considering the actual situation of microbially induced calcium carbonate precipitation, in the process of grouting, the amount of calcium carbonate generated by bacterial liquid is not enough to fill the cracks. According to this situation, PFC particles can be used to distribute sparsely in the cracks, and then the mechanical properties of the simulation results are analyzed according to the uniaxial compression test. It can be concluded that the particles scattered in the cracks cannot play a good role in repair. The overall stress state and crack distribution of the fractured rock mass are the same as those in Fig. 11.

Through the selection of a suitable culture medium environment for the cultivation of *Bacillus pasteurii*, a large amount of calcium carbonate crystals can be induced, which paves the way for the microbial grouting process. In this paper, the variation law of mechanical properties in the process of filling rock fractures with calcium carbonate crystals produced by microbial grouting is mainly described. The linear model is constructed by the particle flow program, and specimens with cracks are generated. Then, the uniaxial compression test before and after calcium carbonate repair is carried out using the parallel bonding model. The strength changes of the specimens are compared, and the operation results of various models are obtained as line graphs.

Figure 12 presents the force–displacement curves obtained from the uniaxial compression simulations before and after calcium carbonate filling. The results show that the peak load increased from approximately 125 kN before filling to about 410 kN after filling, corresponding to a 3.28-fold improvement in load-bearing capacity. This indicates that microbial-induced calcium carbonate precipitation effectively enhances the mechanical properties of fractured rock masses.

### Conclusions and future work

This study systematically investigated the application of MICP for sealing rock fractures through an integrated approach combining laboratory experiments and PFC simulations. The key findings and core innovations are summarized as follows:

#### Systematic optimization of microbial culture conditions

The pH value of the culture medium should be adjusted to the range of 7–10. When the pH value was 9, the bacterial liquid reached the highest biological activity. Considering the cost of rock repair projects, calcium chloride with a lower cost should be selected as the calcium source; the urea concentration of the culture medium should be controlled at 1–1.5 mol/L, the LB concentration adjusted to 20 g/L, and the calcium ion concentration at 1 mol/L to induce the maximum amount of calcium carbonate. When the bacteria were cultured for approximately 20 h, the best mineralization performance could be obtained.

#### Concentration distribution in fractures

The concentrations of urea and urease-producing bacteria are continuously distributed in the fissures, and the concentration changes are also controlled by the mass action rate of chemical reactions.

#### Validation of the minimum filling threshold

In the case of less calcium carbonate after grouting, PFC software simulates the sparse distribution of equivalent generated particles in the fracture. Before and after the pressure test, the load capacity of the specimen does not change significantly, which is almost the same as that of unfilled calcium carbonate, and the repair effect on fractured rock is not significant.

#### Integrated experimental-numerical verification framework

The PFC was used to simulate the filling process of calcium carbonate after microbial grouting by optimizing the bacterial culture conditions. Parameters such as the diameter range, friction coefficient, friction angle, cohesion and stiffness ratio were set to make the mechanical properties of the model the same as those of the actual rock mass. The load strength after repair was 3.28 times higher than that before repair, and the repair effect was good, which allowed the mold to obtain a strong load capacity.

#### Engineering significance and practical guidance

The quantitative results obtained from this study offer actionable insights for practical engineering applications. The 3.28-fold increase in load-bearing capacity (from 125 kN to 410 kN) following MICP treatment provides a benchmark for evaluating repair effectiveness in fractured rock masses. Furthermore, the identification of optimal concentration ranges (urea 1–1.5 mol/L; Ca²^+^ 1 mol/L; pH 9) serves as a reference for designing field-scale grouting protocols. The PFC simulation framework, calibrated with experimental data, can be used to predict the mechanical behavior of treated rock masses under various loading conditions, thereby supporting risk assessment and remediation planning in underground engineering projects such as tunnels, mines, and nuclear waste repositories.

##### Future research directions

###### Adaptability under complex fracture conditions

In the present study, the exploration of microbial grouting mainly focused on relatively simple fracture models. However, in real-world engineering scenarios, rock fractures often present complex characteristics, such as irregular shapes, variable widths, and interconnected networks. Future research should concentrate on the adaptability of microbial precipitation in these complex fracture conditions. The influence of fracture surface roughness on the adhesion and activity of bacteria also needs to be investigated, as rough surfaces may provide more attachment sites for bacteria, but may also impede the flow of nutrient-rich solutions, thereby affecting the precipitation process.

###### Extension to 3D models and different stress environments

The current PFC simulations and experimental set-ups were mostly limited to two-dimensional or simplified three-dimensional scenarios, and the stress environments considered were relatively basic. To better mimic in-situ conditions, future work should extend to comprehensive 3D models. Three-dimensional PFC models can more accurately represent the spatial distribution of particles and fractures in rocks, enabling a more detailed analysis of the interaction between microbial-induced calcium carbonate precipitation and the mechanical behavior of the rock mass. Additionally, different stress environments, such as high-confining pressure in deep-underground engineering or cyclic loading in areas affected by seismic activity, should be incorporated.

###### Integration of chemical reaction kinetics into PFC simulations

As noted in the limitations of this study, the current PFC simulation does not account for the dynamic changes in chemical reaction rates or the progressive generation of calcium carbonate during grouting. To overcome this limitation, future work could integrate chemical reaction kinetics models with the discrete element framework. Coupling PFC with reactive transport models or using multiphysics simulation platforms could enable the simulation of time-dependent urea hydrolysis, ion transport, and mineral precipitation within fractures (33–35). Recent studies have demonstrated the feasibility of such coupled approaches in simulating microbially induced calcite precipitation in porous media (36, 37). Implementing these methods would allow for a more realistic representation of the filling process and provide insights into the optimal injection strategies and fracture-sealing efficiency.

###### Practical challenges and feasibility in engineering applications

Despite the promising results in laboratory and simulation studies, there are several practical challenges when applying microbial grouting technology in engineering. One major challenge is the large-scale production and transportation of bacterial solutions. In engineering projects, a substantial amount of bacterial solution is required to treat large-volume rock masses. Ensuring the viability and activity of bacteria during long-distance transportation and storage is a key issue. Another challenge lies in the control of the microbial-induced precipitation process in the field. Unlike in the laboratory, environmental factors such as temperature, humidity, and the presence of other contaminants in the field can significantly impact the microbial activity and precipitation efficiency. Developing real-time monitoring and control systems to adjust the injection parameters of bacterial solutions and growth substrates according to field conditions is necessary. Future research should aim to develop strategies to address these practical challenges and improve the feasibility of microbial grouting in large-scale engineering applications (38–41).
